# Recent increase in the detection of human parainfluenza virus during the coronavirus disease-2019 pandemic in the Republic of Korea

**DOI:** 10.1186/s12985-022-01938-4

**Published:** 2022-12-12

**Authors:** Heui Man Kim, Jee Eun Rhee, Nam-Joo Lee, Sang Hee Woo, Ae Kyung Park, Jaehee Lee, Cheon Kwon Yoo, Eun-Jin Kim

**Affiliations:** 1 Division of Emerging Infectious Diseases, Bureau of Infectious Diseases Diagnosis Control, Korea Disease Control and Prevention Agency, 187 Osongsaengmyeong2-ro, Osong-eup, Heungdeok-gu, Cheongju-si, Republic of Korea; 2 Bureau of Infectious Diseases Diagnosis Control, Korea Disease Control and Prevention Agency, Cheongju-si, Republic of Korea

**Keywords:** COVID-19 pandemic, Respiratory virus surveillance, Human parainfluenza virus 3

## Abstract

**Background:**

Since the onset of the coronavirus disease-2019 (COVID-19) pandemic, the prevalence of respiratory infectious diseases, particularly, the flu epidemic, has considerably decreased. The low detection rate and decreased number of specimens have hindered the implementation of the Korea Influenza and Respiratory Viruses Surveillance System (KINRESS), a sentinel surveillance system. Most patients with influenza-like illness visit the COVID-19 screening clinic; therefore, the number of samples collected in sentinel surveillance has decreased by more than 50%. Thus, the Korea Disease Control and Prevention Agency supplemented sentinel surveillance with non-sentinel surveillance by private medical diagnostic centers. We report here a delayed and unprecedented high detection of human parainfluenza virus (hPIV) in the Republic of Korea during the COVID-19 pandemic through sentinel and non-sentinel surveillance. We also examined the causes and implications of the changes in prevalence of hPIV.l

**Methods:**

We collected data for 56,984 and 257,217 samples obtained through sentinel and non-sentinel surveillance, respectively. Eight viruses were confirmed using real-time reverse transcription-polymerase chain reaction (PCR) or real-time PCR. Some specimens from the sentinel surveillance were used for genetic characterization of hPIV type 3.

**Results:**

In 2020, hPIV was rarely detected; however, it was detected in August 2021. The detection rate continued to increase considerably in September and reached over 70% in October, 2021. The detection rate of hPIV3 was significantly higher in infants and preschoolers aged 0–6 years in both sentinel and non-sentinel surveillance. Detection of hPIV was delayed in metropolitan areas compared to that in suburban regions. The hemagglutinin-neuraminidase sequences of hPIV3 generated in 2021 were not distinct from those detected prior to the COVID-19 pandemic.

**Conclusions:**

The operation of non-sentinel and sentinel surveillance to monitor respiratory viruses could sensitively detect an unprecedented revival of hPIV in the Republic of Korea during the COVID-19 pandemic.

**Supplementary Information:**

The online version contains supplementary material available at 10.1186/s12985-022-01938-4.

## Background

Since the start of the coronavirus disease-2019 (COVID-19) pandemic, the incidence of respiratory diseases has decreased significantly because of non-pharmacological interventions, including social distancing, following personal hygiene rules, and restrictions on overseas entry [1–3]. The Korea Disease Control and Prevention Agency (KDCA) encountered difficulty in operating the national monitoring system (Influenza and Respiratory Viruses Surveillance System, KINRESS) due to a reduction in respiratory illness-related specimens; however, it sustained national surveillance using the diagnostic results from respiratory patients in private medical diagnostic centers. Since the COVID-19 pandemic began, only non-enveloped viruses, such as human rhinovirus (hRV), human adenovirus (hAdV, and human bocavirus (hBoV), have remained endemic; enveloped viruses, such as influenza virus (IFV) and human respiratory syncytial virus (hRSV), were either not detected or showed very low detection rates in the Republic of Korea [1]. However, human parainfluenza virus (hPIV) was detected in August 2021, and the detection rate increased by more than 50% in the 43rd week. Before the COVID-19 pandemic, hPIV was prevalent mainly during early summer (from May to June) in infants and young children [4]. Here, we report a delayed and unprecedented high detection of hPIV in the Republic of Korea during the COVID-19 pandemic through sentinel and non-sentinel surveillance. We also examined the causes and implications of the changes in prevalence of hPIV.

## Methods

### Sentinel surveillance system

The KDCA has been performing national surveillance for KINRESS since 2000. The system includes 63 clinics and 18 regional laboratories (Public Health and Environment Research Institute, PHERI). The clinic provides up to eight upper respiratory specimens per week; these are collected from patients with influenza-like illnesses, a measured temperature of ≥ 38 °C, and cough with an onset within the past 10 days [5]. PHERI uses commercial respiratory virus detection real-time reverse transcription (RT)-polymerase chain reaction (PCR) or real-time PCR kits (PowerCheck™ Real-time PCR kit series, Kogenebiotech, Seoul, South Korea) to detect IFV (A/H1N1pdm09, A/H3N2, and B), hRSV (A and B), hPIV (1, 2, and 3), human coronavirus (hCoV-OC43, 229E, and NL63), human metapneumovirus (hMPV), hBoV, hAdV, and hRV. The diagnostic results are then reported to the KDCA, which shares the national respiratory virus surveillance data with the public through the National Influenza Center [6].

### Non-sentinel surveillance system

Five private medical diagnostic centers (Seegene Medical Center, Green Cross Laboratory, Eone Laboratory, Seoul Clinical Laboratories, and Samkwang Medical Laboratories) collect respiratory samples from clinics and hospitals across the country. The centers uses commercial respiratory virus detection real-time RT-PCR or real-time PCR kits (Allplex™ Respiratory Panel Assays, Seegene, Seoul, South Korea) to detect IFV (A/H1N1pdm09, A/H3N2, and B), hRSV (A and B), hPIV (1, 2, and 3), hCoV (OC43, 229E, and NL63), hMPV, hBoV, hAdV, and hRV, and report the diagnostic results to the KDCA weekly.

### Clinical specimen collection

A total of 56,984 and 257,217 oropharyngeal or nasopharyngeal swabs were collected from patients through sentinel surveillance (between the 1st week of 2016 and the 47th week of 2021) and non-sentinel surveillance (between the 1st week of 2020 and the 47th week of 2021), respectively. The oropharyngeal and nasopharyngeal swabs were stored in Universal Transport Media (Copan Diagnostics, Inc., Murrieta, CA, USA) before analysis.

### RNA or DNA extraction

Viral RNA or DNA was extracted from 140 µL of transport medium using the MagNA Pure 96 automated extractor (Roche Life Science, Basel, Switzerland) with the DNA and Viral Nucleic Acid Small Volume kit according to the manufacturer’s instructions.

### Genetic analysis of influenza and other respiratory viruses

Commercially available respiratory virus detection real-time PCR kits were used to detect eight respiratory viruses and their subtypes of, including hRSV (types A and B), IFV (type A/H1N1pdm09, A/H3N2, and B), hPIV (type 1, 2, and 3), hCoV (type OC43, 229E, and NL63), hRV, hAdV, hBoV, and hMPV [7]. Viral cDNAs, except those from hAdV and hBoV, were synthesized from 5 μL of extracted nucleic acids using a reverse transcriptase; the reaction mixture was incubated at 50 °C for 30 min, followed by inactivation of the reverse transcriptase at 95 °C for 10 min. PCR amplification was performed with 40 cycles at 95 °C for 15 s and 60 °C for 1 min using an ABI 7500 Fast instrument (Applied Biosystems, Foster City, CA, USA) or a CFX96 real-time cycler (Bio-Rad, Hercules, CA, USA). The amplified PCR products were sequenced by Macrogen (Seoul, South Korea). We used CLC Workbench v X.X (CLC Bio, Aarhus, Denmark) for sequencing and assembly.

### Statistical analysis

The test results of 56,984 sentinel specimens and 257,217 non-sentinel specimens were analyzed; weekly detection rates of eight respiratory viruses (hPIV, IFV, hRSV, hCoV, hRV, hAdV, hBoV, and hMPV) were used for the statistical analysis (Additional file 1). R software (ver. 4.0.2; The R Project for Statistical Computing, Vienna, Austria) was used for most analyses. [8] Statistical significance of the means between two independent groups was analyzed using Welch’s t-test. Statistical significance was set at *P* < 0.05.

### Sequencing and analysis

Five hPIV 3-positive samples were randomly selected each year from 2016 and 2021 for amplification using the primers HPIV3-Seq_6714_sense (5′-AAAGTTACGCAATCCAACTC-3′), HPIV3_Seq_7400_antisense (5′-GAGGTATAAGCATAAATCAGA-3′), HPIV3_Seq_7257_ sense (5′-CACGTCTGGTCTTCCATCT-3′), HPIV3_Seq_7929_ antisense (5′-AACAATGATGGAGTTGACCAT-3′), HPIV3_Seq_7785_ sense (5′-TGGGTATGGAGGTCTTGAAC-3′), and HPIV2_Seq_8577_ antisense (5′-TGTCTATTGTCTGATTGCTGATTA-3′) to generate a 1719-base pair fragment containing the full region of the hemagglutinin-neuraminidase (HN) gene [9]. The amplicons were purified using a QIA PCR purification kit (Qiagen, Hilden, Germany) and sequenced using each sense and antisense primers in both directions on an ABI‐3100 Prism Genetic Analyzer using the BigDye Terminator version 3.1 sequencing kit (Applied Biosystems). The sequences were compared with all available HN sequences of hPIV3 in the National Center for Biotechnology Information (NCBI) Virus annotated specimen collection, taking the date and country name into account. In total, 621 sequences were used to construct a phylogenetic tree, including 25 sequences isolated from the Republic of Korea. Each sequence was aligned using Geneious Prime software (https://www.geneious.com/) and manually trimmed to equal lengths. Temporal phylogenies were inferred in BEAST v1.10.4 [10] using a strict clock, Hasegawa–Kishono–Yano substitution model, and constant coalescent tree prior. Markov chain Monte Carlo chains were run for 100 million steps, with sampling every 10,000 generations. Run convergence was confirmed using Tracer v1.7.1, after 10% burn-in removal [11]. Maximum clade credibility trees were generated using Tree Annotator v1.8 [10] Trees were visualized using FigTree v1.4.4.

### Submission to GenBank

Twenty-five sequences were submitted to GenBank with the full HN region of hPIV3 generated from the samples obtained during the sentinel surveillance. The GenBank accession numbers of the sequences generated in this study are provided in Additional file 2.

## Results

### High detection rate of human parainfluenza virus in sentinel and non-sentinel surveillances before the 2021–2022 season

**Fig. 1:**
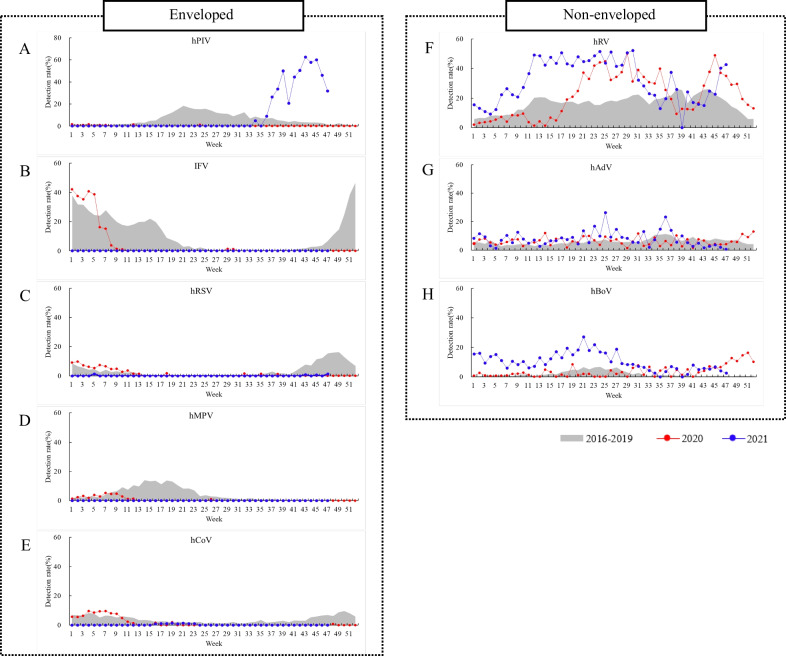
Weekly detection of enveloped and non-enveloped viruses during 2016–2021 through sentinel surveillance. Enveloped viruses: **A** human parainfluenza virus (hPIV), **B** influenza virus (IFV), **C** human respiratory syncytial virus (hRSV), **D** human metapneumovirus (hMPV), and **E** human coronavirus (hCoV). Non-enveloped viruses: **F** human rhinovirus (hRV), **G** human adenovirus (hAdV), and **H** human bocavirus (hBoV)

**Fig. 2:**
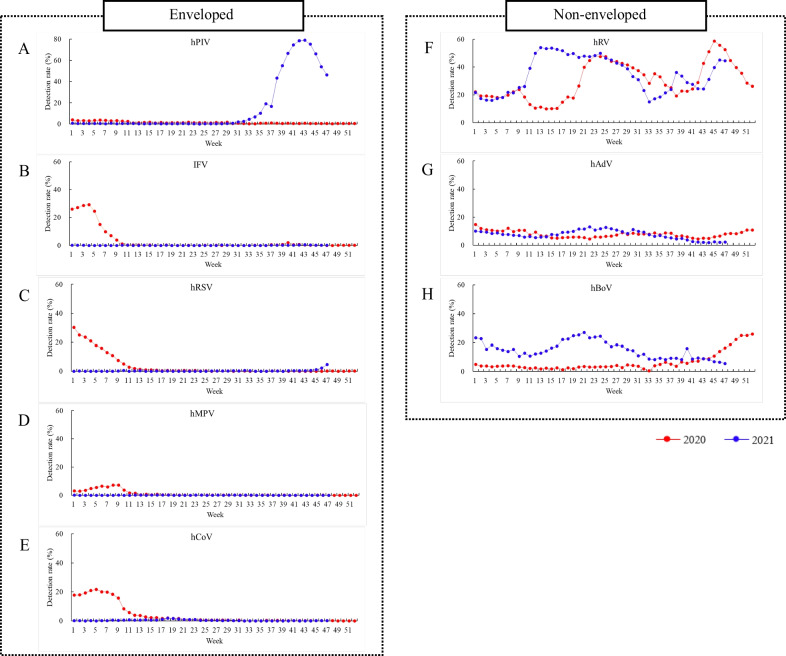
Weekly detection of enveloped and non-enveloped viruses during 2016–2021 through non-sentinel surveillance. Enveloped viruses: **A** human parainfluenza virus (hPIV), **B** influenza virus (IFV), **C** human respiratory syncytial virus (hRSV), **D** human metapneumovirus (hMPV), and **E** human coronavirus (hCoV). Non-enveloped viruses: **F** human rhinovirus (hRV), **G** human adenovirus (hAdV), and **H** human bocavirus (hBoV)

Since the onset of the COVID-19 pandemic in the Republic of Korea, few enveloped viruses (hPIV, IFV, hRSV, hMPV, and hCoV) have been detected in Korea. Respiratory viruses exhibit a seasonality, as observed from their mean weekly detection rate (gray background for 2016–2019 in Fig. 1); however, the COVID-19 pandemic has altered these patterns. Non-enveloped viruses (hRV, hBoV, and hAdV) remained endemic in patients with influenza-like illness. The detection rate of hPIV was 4.9% in the 36th week of 2021, which increased and peaked at 62.5% in the 43th week of 2021; however, other enveloped viruses were not detected or showed very low detection rates (< 1% in hRSV) at the 43rd and 45th week of 2021 (Fig. 1). As the detection rate of hPIV increased, that of hRV decreased. This is the first instance during the 20 years of KINRESS operation in which the detection rate of hPIV was 70%. The detection rate of hPIV increased in the private medical diagnostic centers (non-sentinel), which were included in 2020 and then continued to strengthen the monitoring of respiratory viruses (Fig. 2). hPIV was first detected in the 31st week of 2021, which was 5 weeks earlier than that recorded in KINRESS, and it peaked at the 43rd week at 79.1%, which was the same as that in KINRESS.

### Subtype and age distribution of the detected human parainfluenza viruses

According to a 2016–2019 KINRESS report, the average detection rate of hPIV among the patients with influenza-like illness was 6.24%; among them, hPIV3, hPIV1, and hPIV2 accounted for 59.44%, 27.22%, and 13.34%, respectively. hPIV was detected in all age groups, with the highest detection rate of 62.70% in young children aged 0–6 years (Table 1). Older children and adolescents (7–12 years and 13–18 years old) showed low detection rates of 4.47% and 2.39%, respectively. However, the detection rate in those aged ≥ 19 years (19–48, 50–64, and ≥ 65 years old groups) was approximately 10%, which was higher than that in the older children and adolescents. The detection rate of hPIV was very low (0.41%) in 2020; the detection of type 1 predominated (62.5%) and the prevalence was amongst different age groups was similar to that in 2016–2019, without a higher detection rate in the 7–12 years age group. However, the mean hPIV detection rate in 2021 was significantly higher (13.36%) than that in 2020 (0.41%) and in 2016–2019 (6.24%). A total of 539 hPIV cases were detected in 2021 by the KINRESS, among which 537 belonged to type 3 while the other two were type 2 viruses. The detection rate of type 3 viruses in 2021 by the KINRESS increased considerably compared to that in 2020 and 2016–2019. The detection rate of hPIV in the non-sentinel surveillance was substantially higher in 2021 (27.83%) than in 2020 (1.59%). In 2020, hPIV type 1 was detected at 36.81%, type 2 at 44.83%, and type 3 at 18.35%. In 2021, on the other hand, most of the detected hPIV was type 3 (99.52%). The prevalence was high in the 0–6 years age group in both 2020 and 2021, whereas in the 7–12 years age group, hPIV prevalence was considerably lower in 2021 compared with that in 2020.

**Table 1:** Mean detection rate and subtype distribution in detected human parainfluenza virus and patient age groups

Sentinel/Non-sentinel	Year	Meandetection rate (%)	Subtype distribution (%)			Age group distribution (%)					
			hPIV1	hPIV2	hPIV3	0–6 y	7–12 y	13–18 y	19–48 y	50–64 y	≥ 65 y
Sentinel	2021^*^	13.36^**^ (539/4034)	0.37 (2/539)	0 (0/539)	99.63^**^ (537/539)	92.39^**^ (498/539)	1.86 (10/539)	0.37 (2/539)	2.41 (13/539)	0.93 (5/539)	2.04 (11/539)
	2020	0.41% (24/5820)	62.50 (15/24)	16.67 (4/24)	20.83 (5/24)	50.0 (12/24)	20.83 (5/24)	4.17 (1/24)	8.33 (2/24)	8.33 (2/24)	8.33 (2/24)
	2016–2019	6.24% (2939/47130)	27.22 (800/2939)	13.34 (392/2939)	59.44 (1747/2939)	62.70 (1847/2930)	4.47 (131/2930)	2.39 (70/2930)	10.85 (318/2930)	11.40 (334/2930)	8.19 (240/2930)
Non-sentinel	2021^*^	27.83%^**^ (37,446/134533)	0.18% (65/37446)	0.38% (133/37446)	99.52%^**^ (37,248/37446)	97.15%^**^ (36,354/37419)	1.72% (645/37419)	0.10% (36/37419)	0.37% (138/37419)	0.15% (56/37419)	0.51% (190/37419)
	2020	1.59% (1945/122684)	36.81% (716/1945)	44.83% (872/1945)	18.35% (357/1945)	88.25% (1652/1872)	9.40% (176/1872)	0.69% (13/1872)	0.85% (16/1872)	0.27% (5/1872)	0.53% (10/1872)

^*^2021, 1st–47th week

^**^p < 0.05

hPIV, human parainfluenza virus

### Comparing human parainfluenza weekly detection rate by age groups

**Fig. 3:**
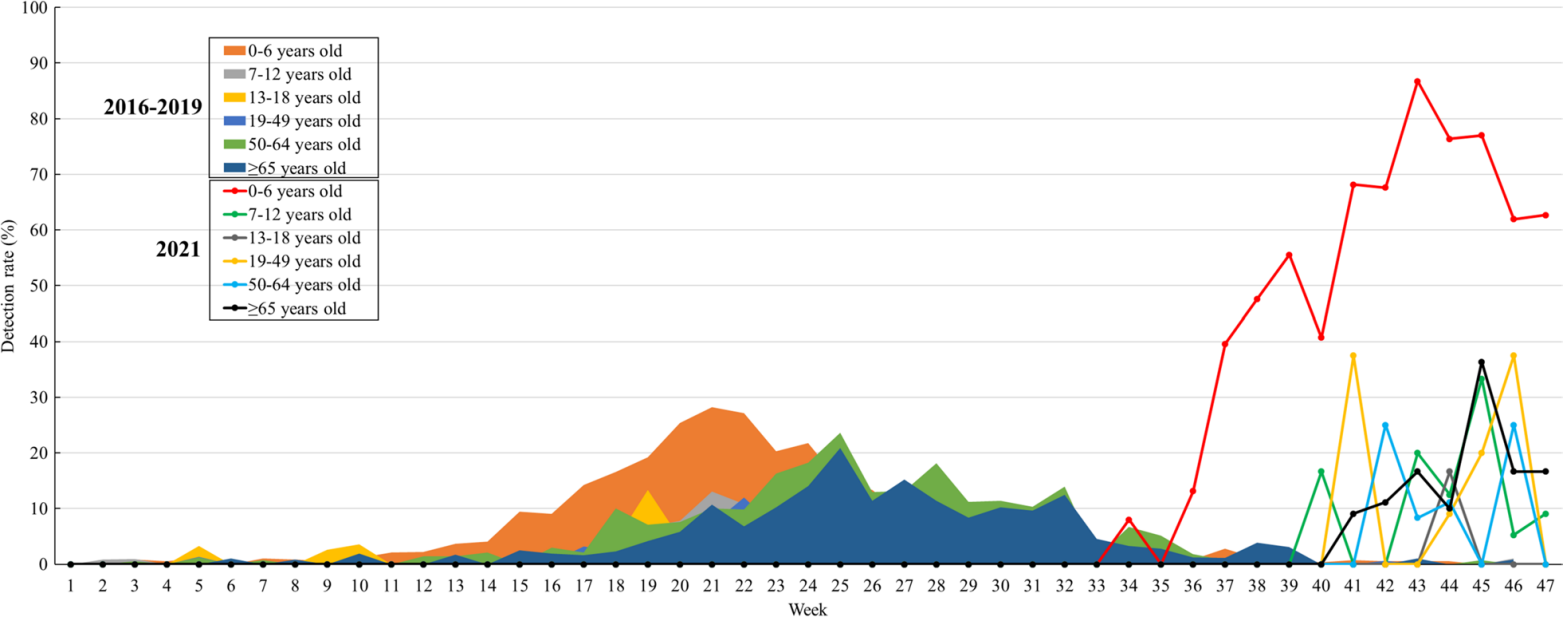
Human parainfluenza virus (hPIV) detection rates in the 2016–2019 and 2021 sentinel surveillance (KINRESS)

The detection rate of hPIV peaked earlier (21st week) in the 0–6 and 7–12 years age groups in the 2016–2019 KINRESS cohort, compared with the other age groups (Fig. 3). The peak detection rate was 28.19% in the 0–6 years group and 13.11% in the 7–12 years group in 2016–2019. The time taken to reach peak detection and the detection rate in the other age groups were as follows, respectively: 24th week and 15.0% in the 13–18 and 19–49 years groups, 25th week and 23.58% in the 50–64 years group, and 25th week and 20.88% in those aged ≥ 65 years of age. In 2021, however, the time to peak detection was delayed in all age groups, and the detection rate increased. There was an unprecedented detection rate of 13.16% in the 34th week in the 0–6 years age group; it gradually increased and reached a peak of 86.75% in the 43rd week. The detection rates were lower and peaked later in the other age groups compared with the 0–6 years age group.

**Fig. 4:**
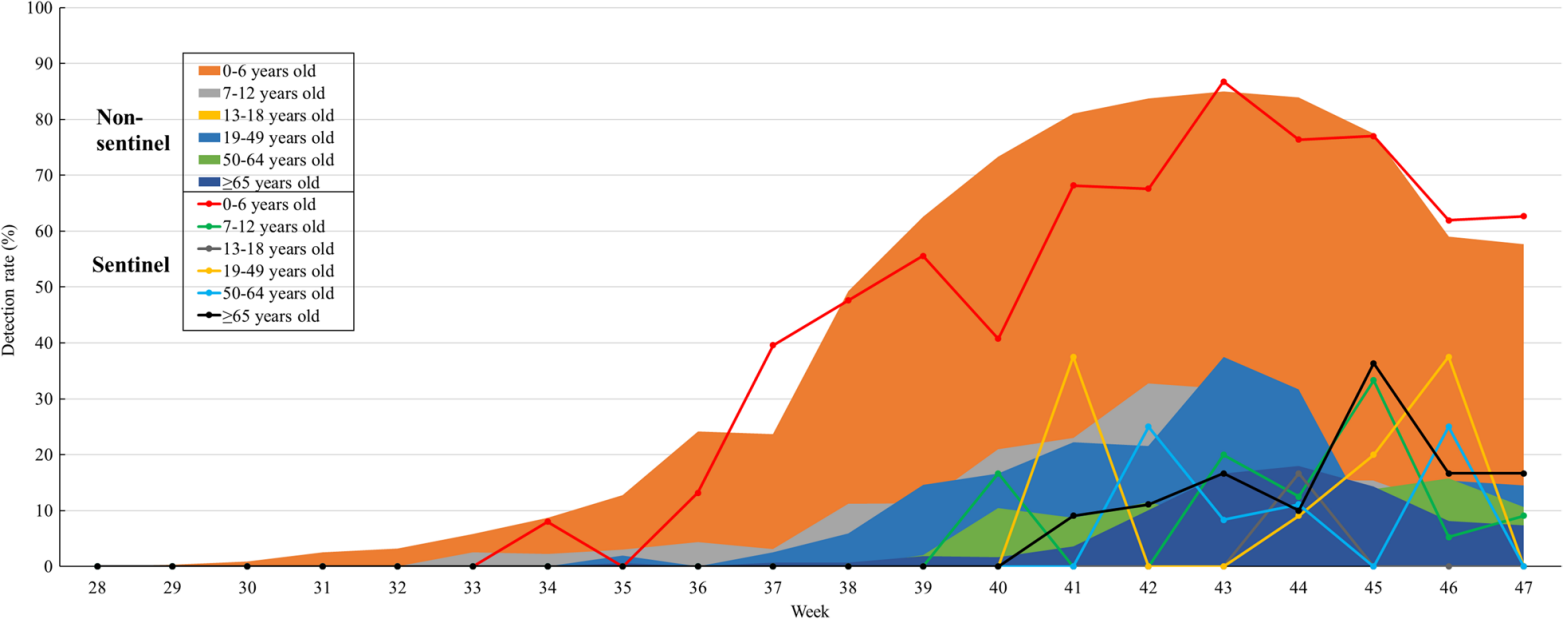
Human parainfluenza virus (hPIV) detection rates by age groups in the sentinel and non-sentinel surveillance

We compared the weekly detection rate between sentinel and non-sentinel surveillance by age group. The initial stages of the epidemic occurred earlier in the non-sentinel surveillance compared with that in the sentinel surveillance (Fig. 4). In the non-sentinel surveillance, hPIV was initially detected in the 0–6 years group at 0.87% in the 30th week; the detection rate increased gradually, and peaked (84.96%) in the 43rd week, similar to that observed in the sentinel surveillance. The detection rate of hPIV decreased in all age groups in the 43rd week in the non-sentinel surveillance group.

### Comparing human parainfluenza weekly detection rate by geography in non-sentinel surveillances

**Fig. 5:**
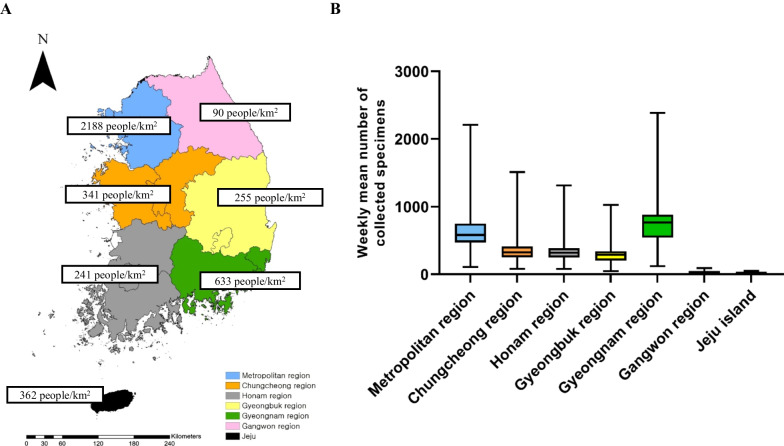
Population density and number of specimens by region in the Republic of Korea. **A** Geographic distribution and the population density of each region. **B** The Box and Whisker Plot for weekly mean number of collected specimens by region

**Fig. 6:**
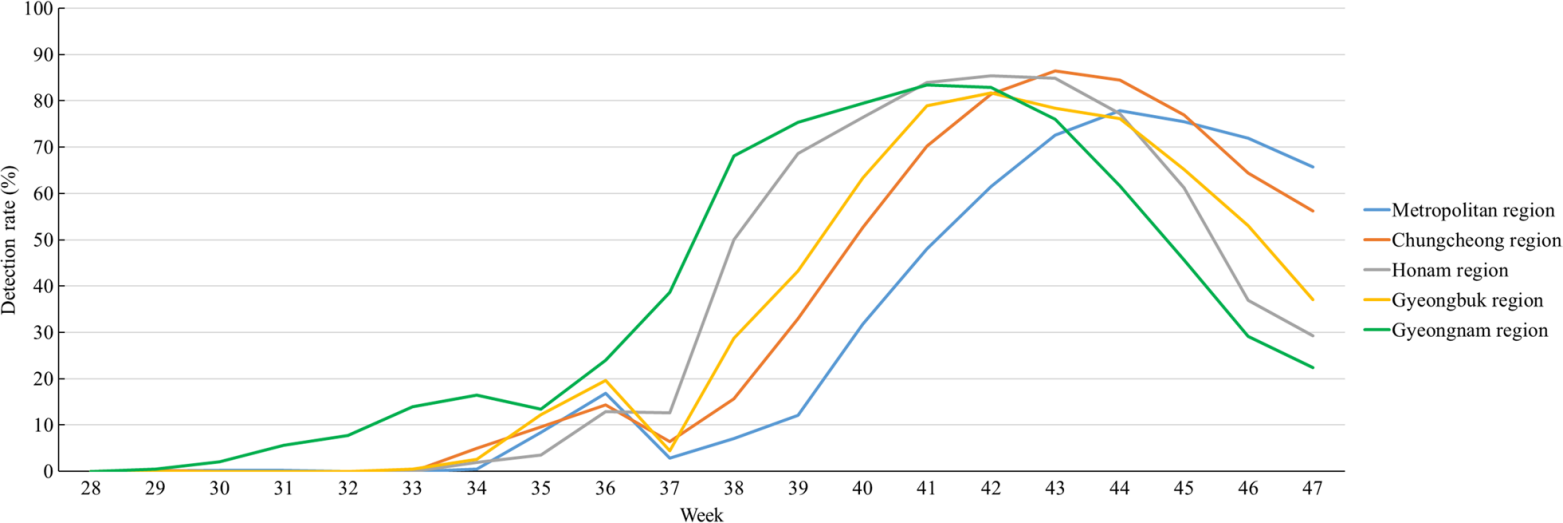
Weekly detection rate of human parainfluenza virus 3 by region in the Republic of Korea

The Republic of Korea is divided into six regions and one island: Metropolitan, Chungcheong, Honam, Gangwon, Gyeongbuk, and Gyeongnam regions and Jeju Island (Fig. 5A). The population density is the highest (with 2188 people/km^2^) in the metropolitan region. Gyeongnam region is the second most populated, with 633 people/km^2^. The mean number of specimens collected per week was the highest in the Gyeongnam region (919), followed by the metropolitan region (878) (Fig. 5B). The number of specimens from the Gangwon region and Jeju Island was less than 30; therefore, these were excluded from this analysis. The hPIV detection rate from the five regions of the Republic of Korea was compared based on the data from the non-sentinel surveillance (Fig. 6). hPIV detection was first noted in the 30th week in the Gyeongnam region and peaked in the 41st week, which was earlier than in other regions. hPIV was detected in most regions, except the metropolitan region: detection in the metropolitan region started in the 35th week and peaked in the 44th week, which was delayed compared to that in the other regions.

### Comparison of the genetic characteristics of the human parainfluenza virus prevalent in 2016–2021 in sentinel surveillance

**Fig. 7:**
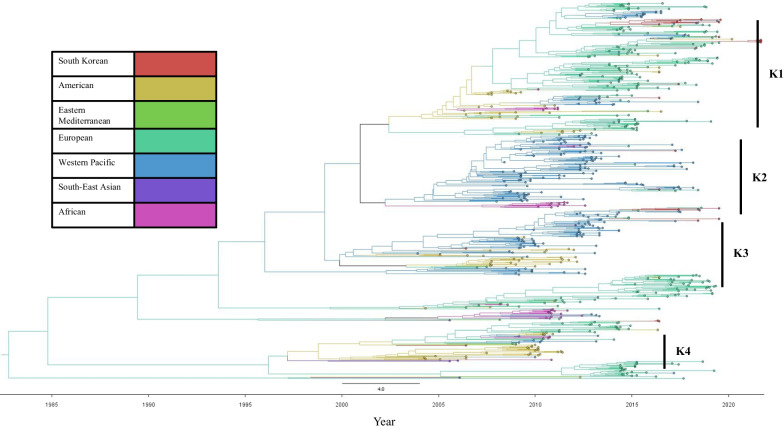
Time-measured phylogenetic tree of the human parainfluenza virus 3 (hPIV3) hemagglutinin-neuraminidase gene. The geographical regions are colored based on where the virus was isolated

We investigated the genetic characteristics of hPIV type 3 detected over six years (2016–2021) in KINRESS. Five respiratory specimens from each year were randomly selected to sequence the major surface glycoprotein HN. We reconstructed Bayesian phylogenetics using the full-length HN gene (1719 base pairs) of hPIV3 and publicly available sequences (N = 650) with confirmed sample collection date and country, obtained from NCBI. hPIV3 sequenced from the Republic of Korea could be divided into four clades: K1, K2, K3, and K4 (Fig. 7). K1 wass the major clade of hPIV3 detected since 2016, includinghPIV3 detected in Germany and USA. However, HN sequences generated from the 2021 samples were relatively similar to those generated from the 2016–2019 samples.

## Discussion

Since the first case of COVID-19 reported on January 20, 2020, in the Republic of Korea, [8] the disease has spread nationwide with several distinct epidemic waves [12–14]. The recent wave in the Republic of Korea was different from the previous waves, with a larger number of confirmed cases infected with the delta variant [15, 16] and there were reports of breakthrough infections [17]. The implementation of high-intensity social distancing and isolation measures since the first wave of COVID-19 affected the detection rate of the respiratory virus. Non-enveloped viruses (hRV, hAdV, and hBoV) remained endemic, whereas enveloped viruses (IFV, hRSV, hMPV, and hCoV [229E, NL64, and OC43]) were rarely detected in sentinel and non-sentinel surveillance in the Republic of Korea [1]. The detection of hPIV has been extraordinarily high in both surveillance systems since the 43rd week of 2021. In contrast to the previous year, the relaxation in social distancing, owing to the high rate of COVID-19 vaccination, has resulted in increased movement between the regions; childcare facilities (care centers and kindergartens) and schools (elementary, middle, and high schools) are operating normally through offline classes; and most people are engaging in economic activities. These changes resulted in an unprecedented increase in the detection rate of hPIV, especially in the young age groups, in September 2021 in the Republic of Korea. Among the enveloped viruses, hPIV is the most common pathogen causing common cold in infants [18, 19]. Most children over 5 years of age possess antibodies against hPIV3 [20, 21]; however, infants are vulnerable to hPIV3 infection [22, 23]. Therefore, the detection rate of hPIV is higher in infants, especially with an increase in type 3 detection from the zero-base during the COVID-19 pandemic. These changed patterns were also observed in the National Respiratory and Enteric Virus Surveillance System in the USA [24]. The detection rate of hPIV in the USA was low in 2020; however, it peaked in June, 2021. This indicates a delayed revival, considering earlier patterns [25]. The detection rate of hPIV in Hong Kong showed an unprecedented increase in November 2021 [26].

In the case of hRSV, delayed and surged detection was reported in several countries [27–30]. While hRSV was not detected in the sentinel surveillance in 2021 in the Republic of Korea, it was detected at low levels in the non-sentinel surveillance since the 45th week of 2021. Prior to the start of the COVID-19 pandemic, hRSV was generally prevalent in autumn and early winter, followed by the hPIV epidemic in early spring and summer in the Republic of Korea. Therefore, the hRSV detection rate in the non-sentinel surveillance exhibited a delayed high prevalence in late 2021, similar to that observed for hPIV. The discrepancy between the number of hPIV cases detected and the number of patients in all the age groups could be attributed to coinfection among the hPIV types. The majority of coinfections involved hPIV3 and hPIV1 or hPIV2 in the 0–6 years age groups.

As a sentinel surveillance tool, the KINRESS has an advantage; it enables comparison of the trends in the detection of respiratory viruses because it includes over 20 years of accumulated data from the same clinics in the Republic of Korea. However, the number of respiratory specimens in the KINRESS decreased by 50% during the COVID-19 pandemic [31]. To overcome the limitation posed by the reduction in the number of respiratory specimens, the KDCA supplemented sentinel surveillance by analyzing the results of the non-sentinel diagnostic tests from private medical diagnostic centers, which conduct thousands of tests (mean = 2596) per week for diagnosing respiratory patients. In the non-sentinel surveillance, the first case of hPIV occurred 4 weeks earlier, and the detection rate was more stable than that in the sentinel surveillance system. To strengthen respiratory surveillance, operating both sentinel and non-sentinel surveillance is effective. The delay in the parainfluenza epidemic in the metropolitan region, despite the highest population density, could be attributed to the high incidence of COVID-19 and the subsequent strengthened social distancing. In the other regions, social distancing and quarantine measures were eased compared to those in the metropolitan region; this could have led to the faster spread of the parainfluenza epidemic. In particular, the detection of hPIV in the Gyeongnam region, which has the highest population density among the non-metropolitan areas, started in the 30th week and peaked in the 43rd week, which was faster by 5 and 3 weeks, respectively, than that in the metropolitan region.

The genetic characteristics of the revived hPIV in 2021 from the Republic of Korea were not distinct from those of the previously prevalent hPIV. Among the hPIV types, type 3 was detected predominantly, and most were confirmed as the K1 clade, which is the major clade detected every year in the Republic of Korea. We could not identify any molecular determinants in the HN sequences of the revived hPIV. The COVID-19 pandemic led to the strengthening of social distancing and quarantine policies worldwide in 2020 and eventually resulted in a very low incidence of previously prevalent infectious diseases associated with both enveloped and non-enveloped viruses [32–34]. However, in 2021, as social distancing was eased, owing to the improvement in COVID-19 vaccination coverage, the revival of respiratory viruses at unusual times was reported in some countries [25, 35, 36]. Among the enveloped viruses, hPIV was the first to be revived in the Republic of Korea with delayed and unprecedented high (over 70%) detection rates. These changes in the prevalence of respiratory viruses indicate that multiplex diagnosis of other respiratory viral infections, as well as COVID-19, is needed to detect these viruses at unusual times [25]. The increasing incidence of hPIV suggests that other respiratory infectious diseases that were dormant during the COVID-19 pandemic are gradually reviving, without following previous seasonality. Social distancing prevents the spread of COVID-19; it is crucial to habituate personal hygiene rules, such as wearing a mask, hand washing, and practicing cough etiquette, to prevent respiratory infectious diseases as well as COVID-19. Children, pregnant women, and the elderly should be vaccinated to reduce the anticipated morbidity and mortality. This study focused on surveillance data; hence, the analysis for clinical relevance was limited, and further studies on the clinical manifestations, complications, and treatment of hPIV-infected patients are needed.

## Supplementary Information

**Additional file 1**. Weekly detection rates of eight respiratory viruses were used for statistical analysis. Human parainfluenza virus (hPIV), influenza virus (IFV), human respiratory syncytial virus (hRSV), human coronavirus (hCoV), human rhinovirus (hRV), human adenovirus (hAdV), human bocavirus (hBoV), and human metapneumovirus (hMPV)

**Additional file 2**. GenBank accession numbers of the sequences generated in this study. GeneBank accession numbers for full-length hemagglutinin-nueraminidase gene (1719 base pairs) of twenty-five hPIV3 used in genetic analysis.

## Data Availability

All data generated or analyzed during this study are included in this published article and its supplementary information files.

## Abbreviations

COVID-19: Coronavirus disease-2019
hAdV: Human adenovirus
hBoV: Human bocavirus
hCoV: Human coronavirus
hMPV: Human metapneumovirus
hPIV: Human parainfluenza virus
hRV: Human rhinovirus
hRSV: Human respiratory syncytial virus
IFV: Influenza virus
KDCA: Korea disease control and prevention agency
KINRESS: Korea influenza and respiratory viruses surveillance system
